# Real-life evidence of low-dose mepolizumab efficacy in EGPA: a case series

**DOI:** 10.1186/s12931-021-01775-z

**Published:** 2021-06-23

**Authors:** Aikaterini Detoraki, Eugenio Tremante, Remo Poto, Emanuela Morelli, Giuseppe Quaremba, Francescopaolo Granata, Antonio Romano, Ilaria Mormile, Francesca Wanda Rossi, Amato de Paulis, Giuseppe Spadaro

**Affiliations:** 1 Department of Internal Medicine, Clinical Immunology, Clinical Pathology and Infectious Diseases, Azienda Ospedaliera Universitaria Federico II, Naples, Italy; 2 Division of ENT, Azienda Ospedaliera Dei Colli, Naples, Italy; 3 Post Graduate Program in Clinical Immunology and Allergy, University of Naples Federico II, Naples, Italy; 4 Department of Advanced Biomedical Sciences, University of Naples Federico II, Naples, Italy; 5 Department of Neurosciences, Reproductive and Odontostomatological Sciences, Maxilofacial Surgery Unit, University of Naples Federico II, Naples, Italy; 6 Department of Translational Medical Sciences, University of Naples Ferderico II, Naples, Italy; 7 Allergy and Clinical Immunology Center for Basic and Clinical Immunology Research (CISI), University of Naples Federico II, Naples, Italy

**Keywords:** Eosinophilic granulomatosis with polyangiitis, Severe eosinophilic asthma, Chronic rhinosinusitis, Nasal polyposis, Mepolizumab

## Abstract

Eosinophilic granulomatosis with polyangiitis (EGPA) is a rare, small vessel, necrotizing vasculitis. The disease is mainly characterized by hypereosinophilia and asthma with frequent sinonasal involvement, although multiple organs can be affected, including the heart, lungs, skin, gastrointestinal tract, kidneys, and nervous system. IL-5 production is pathogenetically central for the development of the disease by promoting proliferation, transvascular migration and functional activation of eosinophils. The degree of blood and tissue eosinophilia appears to be associated with disease pathogenesis and eosinophil depletion represents a promising treatment approach for EGPA. We prospectively evaluated the efficacy and safety of a low dose (100 mg q4w), 12-month course of mepolizumab, an anti-IL-5 monoclonal antibody, in eight patients with severe asthma and active EGPA. Patients were recruited by the tertiary care center of Clinical Immunology and Allergy, University of Naples Federico II. The following outcomes were assessed before (T0), and after 6 (T6) and 12 months (T12) of mepolizumab treatment: Birmingham Vasculitis Activity Score (BVAS), prednisone intake, Sino-Nasal Outcome Test (SNOT-22), Total Endoscopic Polyp Score (TENPS), Asthma Control Test (ACT), Forced Expiratory Volume one second (FEV1)%, blood eosinophilia. BVAS score significantly decreased showing a sharp reduction in disease activity score. Clinical improvements in terms of sinonasal scores and asthma symptoms were observed, in parallel with a drastic drop in eosinophil blood count. Prednisone intake was significantly reduced. In two patients, asthma exacerbations led to discontinuation in mepolizumab therapy after 6 and 12 months despite BVAS reduction. Mepolizumab treatment was well tolerated, and no severe adverse drug effects were registered. In conclusion, our 12-month real-life study suggests that mepolizumab may be beneficial and safe in active EGPA patients by improving disease activity score, sinonasal and asthma outcomes while reducing the burden of prednisone intake.

## Introduction

Eosinophilic granulomatosis with polyangiitis (EGPA), formerly Churg-Strauss syndrome, is a rare disease characterized by potentially life-threatening systemic, small vessel, eosinophilic vasculitis [1, 2]. Prevalence in the general population is 10.7–13/million, with an annual incidence estimated as 1–4/million/year [3]. The incidence is higher among asthmatics, ranging from 34.6 to 64.4/million/year [4–6]. All EGPA patients have hypereosinophilia with asthma and pulmonary involvement being present in 86%, chronic rhinosinusitis with nasal polyposis (CRSwNP) is observed in 29% of these. Multiple organs can be affected by EGPA, including the heart, lungs, skin, gastrointestinal tract, kidneys, and nervous system. The mean age at diagnosis is 48 years without a predominance of sex [7, 8].

EGPA pathophysiology remains only partly understood; it is associated with anti-neutrophilic cytoplasmic antibodies (ANCAs), usually directed against myeloperoxidase (MPO) or proteinase 3 (PR3) in approximately 40% of the patients [9, 10]. ANCA status may negatively influence disease presentation and ANCA-positive patients should be treated more aggressively [11]. The degree of blood and tissue eosinophilia appears to be associated with disease pathogenesis; therefore, eosinophil depletion represents a promising treatment approach for EGPA.

Interleukin 5 (IL-5) is a major hematopoietin regulating the life cycle of eosinophils [12, 13]. Circulating levels of IL-5 are increased in EGPA patients, probably released by human peripheral blood mononuclear cells (PBMCs) through T-cell activation [12–14]. IL-5 promotes eosinophil adhesion to vascular endothelium and CC chemokine receptor 3 dependent migration of eosinophils from the vasculature [15]. Thus, IL-5 production in EGPA is pathogenetically central for the development of the disease.

EGPA management is based on reducing active inflammation, modulating the immune response and managing disease-specific and/or treatment-related complications. In a proportion of patients, remission may be achieved with systemic corticosteroid therapy, alone or in combination with more potent immunosuppressant therapies (e.g., cyclophosphamide, methotrexate, azathioprine, mycophenolate mofetil) but a relapse rate of 30–40% is reported [16]. Moreover, patients receiving long-term corticosteroid and immunosuppressive therapy may experience severe adverse effects including osteoporosis, diabetes, hypertension increased risk of infection and weight gain. Therefore, the main target in the treatment of EGPA is to induce long-term remission and reduce the burden of systemic corticosteroids and immunosuppressors: in this contest, recent studies demonstrated potential advantages of the use of monoclonal antibodies targeting IgE and IL-5 pathways in EGPA [17–19].

Mepolizumab is a fully humanized monoclonal antibody that binds to free IL-5 with high specificity and affinity, thus preventing the interaction to the alpha chain of IL-5 receptor (IL-5R) on the eosinophilic cell surface. The drug is approved as add on treatment of patients with severe eosinophilic asthma (SEA) accordingly to the GINA guidelines and it is administered subcutaneously (s.c.) every four weeks with a dosage of 100 mg (100 mg q4w) [20, 21].

Previous studies demonstrated efficacy and safety of higher dosages of mepolizumab (300 mg q4w) in the induction and maintenance of remission of relapsing or refractory EGPA [19, 22–24]. Only a small number of real-life studies evaluated the efficacy of low, “asthma-tailored” dose of mepolizumab in EGPA patients [25–27].

The aim of this study was to investigate the effects of low-dose mepolizumab (100 mg q4w, s.c.) in EGPA patients in terms of disease control [evaluated by the Birmingham Vasculitis Activity Score (BVAS)], sinonasal control [by using the Sino-Nasal Outcome Test (SNOT-22) score and Total Endoscopic Polyp Score (TENPS)], and asthma control [by Asthma Control Test (ACT) and Forced Expiratory Volume at 1 s % (%FEV1: FEV1/FEV1 predicted)], after 6 (T6) and 12 (T12) months of treatment. In addition, reduction of eosinophil blood count and oral prednisone intake were also evaluated at T6 and T12.

## Materials and methods

The study involved the Division of Clinical Immunology and Allergy, a tertiary care center of the University of Naples Federico II. Patients recruited in the study were in follow-up in the outpatient clinic for EGPA and comorbid SEA. Head and neck consultation was performed by an Ear, Nose and Throat (ENT) specialist and a maxillo-facial surgeon. Inclusion criteria were diagnosis of EGPA according to the American College of Rheumatology 1990 criteria [28] and/or the definition of EGPA adopted by the 2012 Chapel Hill Consensus Conference [29]. The key exclusion criteria were pregnancy, malignancies, immune-deficiencies, other hypereosinophilic syndromes or other ANCA vasculitis, allergic bronchopulmonary aspergillosis and *Strongyloides stercoralis* infection.

Patients were treated with mepolizumab as add-on therapy to oral corticosteroids (OCS), inhaled long-acting beta_2_-agonists (LABA)/corticosteroids (ICS) associations, plus long-acting anti-antimuscarinic drugs (LAMA) as stated by step 5 GINA guidelines for severe asthma, and intranasal corticosteroids (mometasone 50 μg spray: one spray in the morning and one spray in the evening for each nostril) for rhinosinusitis with or without nasal polyposis. For one out of eight patients of the study not receiving prednisone at baseline, mepolizumab was added to immunosuppressor treatment. As mepolizumab is not yet approved for EGPA in Italy, the drug was prescribed for treatment of severe eosinophilic asthma, according to the recommendations of the Italian Drug Agency (AIFA) at a dosage of 100 mg q4w s.c. for a 12-month course.

The parameters used to verify the efficacy of mepolizumab were: BVAS, SNOT-22, TENPS, ACT, FEV1%, blood eosinophils and prednisone dosage, performed at baseline (T0) and after 6 (T6) and 12 months (T12) of mepolizumab treatment.

The BVAS is designed to document clinical features that are directly due to active vasculitis. It rates 10 areas of interest (general, cutaneous, mucous membranes, eyes, ENT, chest, cardiovascular, abdominal, renal, nervous system, other) [30]. Disease manifestations are scored only when they are attributable to active vasculitis. Score superior to 0 suggests active disease.

SNOT-22, the most commonly used instrument for patients reported outcomes [31, 32] was administered. It rates 22 different symptoms from 0 (no problem) to 5 (problem as bad as it can be) related to rhinological, ear, facial, general, physical, and psychological domains. Rhinofibroscopy (with a straight forward telescope 0° and forward oblique telescope 30°, diameter 4 mm, length 18 cm. Straight forward telescope 0°, diameter 3 mm, length 14 cm) was performed in order to validate a diagnosis of CRSwNP [33]. Each nostril was scored as follows: score 0-no polyps; score 1-small polyps in the middle meatus not reaching below the inferior border of the middle concha; score 2-polyps reaching below the lower border of the middle turbinate; score 3-large polyps reaching the lower border of the inferior turbinate or polyps medial to the middle concha: score 4-large polyps causing almost complete congestion/nasal obstruction of the inferior meatus. A patient with severe bilateral polyposis would therefore have a maximum score of eight. Sinus involvement was also assessed by computer tomography scan and/or magnetic resonance imaging.

ACT is a validated five-item questionnaire for assessing asthma control. Each item is scored on a five-point scale from 1 to 5 (1 = worst; 5 = best), with a total score of 5–19 and 20–25 points describing uncontrolled and well-controlled asthma, respectively.

Spirometry (model "Quark PTF", COSMED) before and after salbutamol 400 mcg, was performed according to the guidelines of the American Thoracic Society/European Respiratory Society (ATS/ERS) [34]. FEV1, Forced Vital Capacity (FVC), FEV1/FVC were measured, and the best of three forced maneuvers was recorded. Results were expressed both as absolute values and as a percentage of the predicted values referred to European Respiratory Society (ERS) 1993 reference values.

Asthma exacerbations were registered as an acute or subacute worsening in symptoms and lung function from the patient’s usual status [35].

Pulmonary involvement was assessed by high-resolution tomographic chest examination based on the presence of ground-glass opacities, areas of consolidation with thinning of the bronchial wall, and the presence of small centrilobular nodulations or pleural effusion. Cardiac involvement, a negative prognostic factor for EGPA, was assessed by electrocardiographic and echocardiographic study. The presence of neurological interest (mononeuropathy and polyneuropathy) was confirmed with clinical examination and electromyography. Kidney and liver were evaluated through laboratory tests and abdomen ultrasound. Peripheral blood eosinophil counts were measured using an automated hematology analyzer. Prednisone intake (mg/day) was registered during scheduled visits.

Treatment with immunosuppressors and /or prednisone was managed by the same physicians that prescribed mepolizumab. From week 4 post baseline onward, in those patients who showed improvement in respiratory symptoms and lack of manifestations attributable to active vasculitis, prednisone was tapered downwards according to the standard of care practice (i.e., 2.5 mg reduction every 2 weeks).

Safety was clinically monitored on the basis of the frequency and severity of adverse events (hypersensitivity reactions, local site reactions, headache, respiratory and urinary tract infection, nasal congestion, eczema, and back pain).

All procedures complied with the Helsinki Declaration of 1964, subsequently revised in 2013. The study protocol was approved by the ethical committee of the University of Naples Federico II. Informed consent was obtained from all patients who agreed to participate to this study.

## Statistical analysis

Data as demographic, clinical, and other disease-related variables were statistically described with the use of frequencies (percentages when appropriate) for categorical variables, and the mean and SD for quantitative variables. The comparison between the sample means at T0 vs. T6, T6 vs. T12, and T0 vs. T12, for the variables of the continuous type, was performed by means of Student’s t-test, by calculating the 95% confidence interval. All p values were two-sided, and values less than 0.01 were considered significant. The calculations were performed using IBM SPSS Statistics, v.20.0 software (IBM Corp. Armonk, NY).

## Results

8 patients (6 men, 2 women) mean age 55.75 years, were included in the study. Table 1 shows patient’s characteristics at baseline (T0) before mepolizumab treatment. All patients had active EGPA as BVAS at T0 was 21.7 ± 6.4 and severe eosinophilic asthma (mean ACT was 8.3 ± 2. and mean % FEV1 was 73 ± 24.5) with chronic rhinosinusitis with (CRSwNP) or without nasal polyps (CRSsNP) (mean SNOT-22 at T0 was 49 ± 22.9). At T0, nasal polyposis was present in 4 of the patients: mean TENPS at T0 was 3.4 ± 3.8 while 7 patients had already been treated by functional endoscopic sinus surgery (FESS). 7 patients were on OCS (median prednisone: 16.7 ± 9 mg/day); five patients had previously received or were on treatment with immunosuppressant drugs (cyclophosphamide, azathioprine, methotrexate, mycophenolate mofetil) and two had previously received anti-IgE (omalizumab) treatment. High mean blood eosinophilia (2.384 ± 2.210 cells/μL) was registered at baseline.

**Table 1:** Characteristics of patients at baseline (before mepolizumab initiation)

Age (y), no	8
mean ± SD	55.75 ± 13.13
Sex, no	8
Male, no. (%)	6
Female, no. (%)	2
Age at diagnosis (y)	51.50 ± 12.60
BMI (Kg/m^2^)	24.92 ± 4.10
Asthma	8
ENT involvement	8
Polyposis, no	8
Yes, no. (%)	7
No, no. (%)	1
Rhinosinusitis, no	8
Yes, no. (%)	8
Yes, no. (%)	0
SNOT-22	
Mean ± SD	49 ± 22.9
TENPS no	8
Mean ± SD	3.4 ± 3.8
Polyposis surgery, no	8
Yes, no. (%)	7
No, no. (%)	1
Eosinophils (cell/mcl),	
Mean ± SD	2.384 ± 2.209
BVAS	
Mean ± SD	21.75 ± 6.41
ACT	
Mean ± SD	8.3 ± 2.7
FEV1%	
Mean ± SD	73 ± 24.5
Prednisone intake	
Mean ± SD	16.7 ± 9
Pulmonary infiltrates	5
Peripheral nervous system involvement	3
Arthralgia/arthritis	6
Cardiac involvement	1
Glomerulonephritis	0
Ocular involvement	1
Skin involvement	4
ANCA positivity patients	2
Present and Previous treatments	
OCS	7
Azathioprine	2
Intravenous cyclophosphamide	2
Methotrexate	0
Mycophenolate mofetil	2
Omalizumab	2

*BMI* Body mass index, *SNOT-22 *Sino-Nasal Outcome Test, *TENPS* Total endoscopic polyp score, *BVAS* Birmingham vasculitis activity score, *ACT* Astma control test, *FEV1% *forced expiratory volume at 1 s (%), *ANCA* antineutrophil cytoplasm antibodies, *OCS* oral corticosteroids

**Fig. 1:**
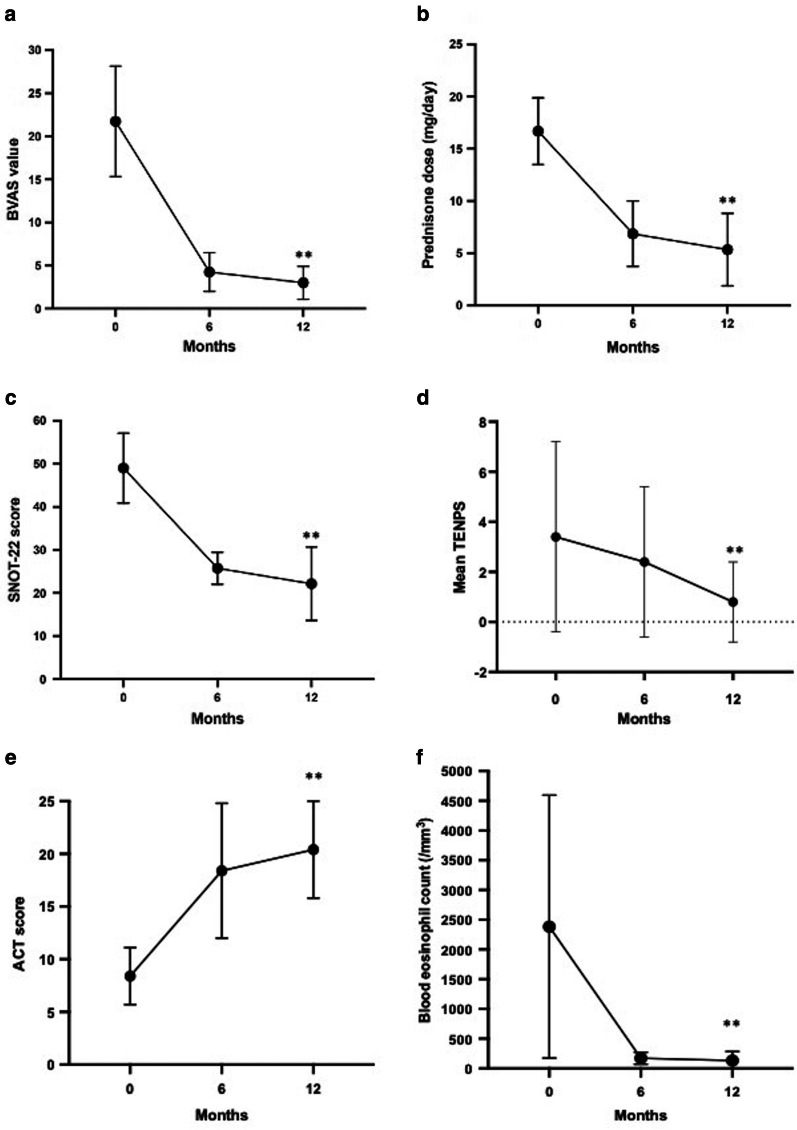
Parameters used to verify the efficacy of mepolizumab treatment at T0, T6 and T12. Birmingham Vasculitis Activity Score (BVAS) (**a**). Prednisone intake (mg/day) (**b**). Sino-Nasal Outcome Test (SNOT-22) (**c**). Total endoscopic nasal polyp score (TENPS) (**d**). Asthma Control Test (ACT) (**e**). Blood eosinophil count (cells/μl) (**f**)

Measurements were performed according to study protocol at 6 and 12 months of mepolizumab treatment. Interestingly, BVAS significantly decreased to 3.6 ± 1.3 at T6 and 3.0 ± 1.9 at T12 (T0 to T12 p < 0.01) (Fig. 1a) suggesting an overall improvement in vasculitic associated manifestations (heart, lungs, skin, gastrointestinal tract, kidneys, and nervous system). Oral prednisone was reduced during the study period: mean prednisone at T6 was 7.1 ± 9.5 mg/day and was 5.3 ± 9.1 mg/day at T12 (T0 to T12 p = 0.03) (Fig. 1b). A significant clinical improvement was reported for sinonasal disease as mean SNOT-22 was 26.2 ± 11.3 at T6 and 22.1 ± 22.5 at T12 (T0 to T12 p < 0.01) (Fig. 1c); in addition, a decrease in TENPS was observed at T6 (2.4 ± 3) and T12 ( 0.8 ± 1.6) (T0 to T12, p = 0.06) (Fig. 1d).

**Fig. 2:**
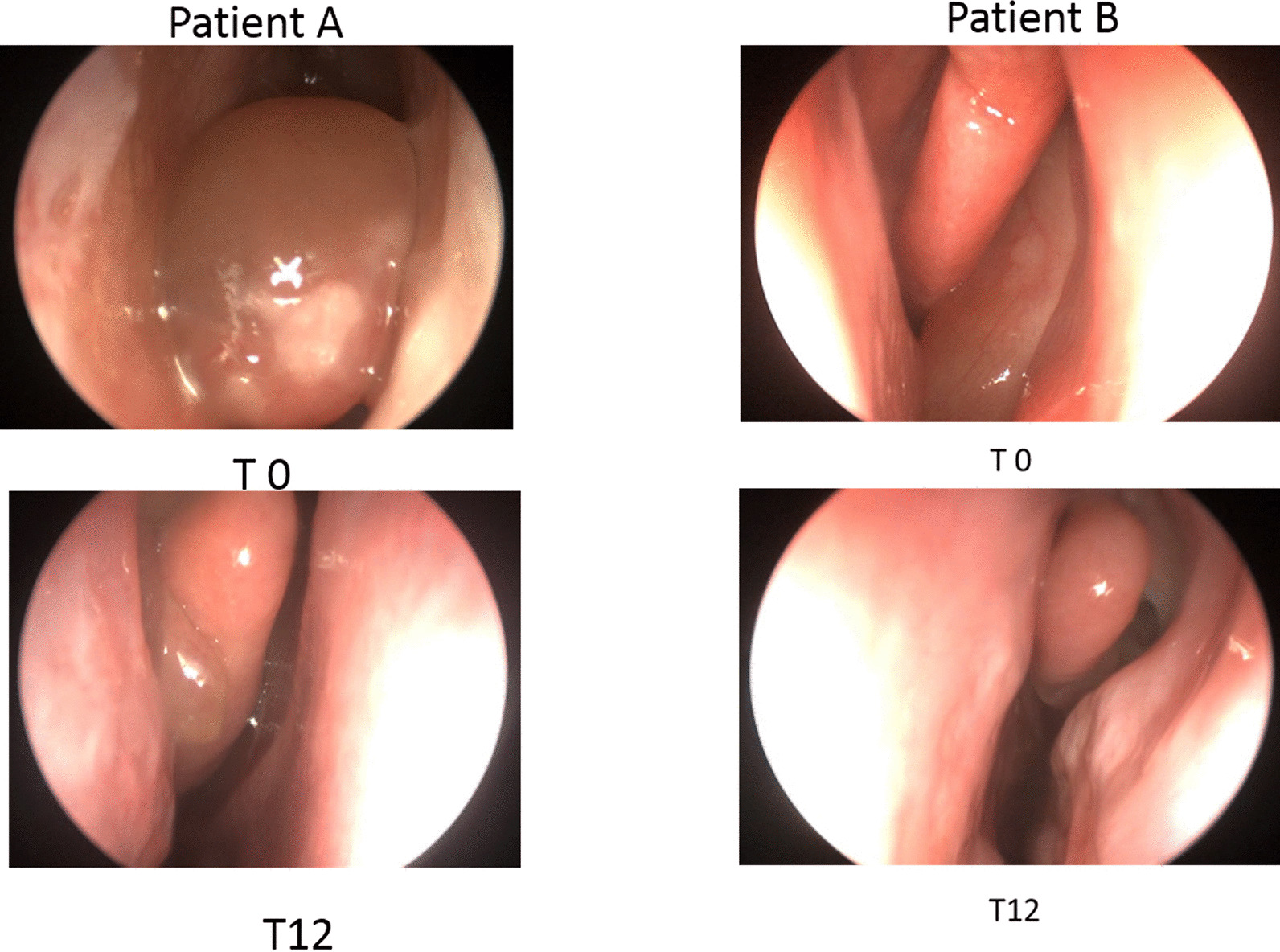
Rhinofibroscopy findings of two patients (**A** and **B**) before (T0) and after 12 months of mepolizumab treatment (T12). Patient A: TENPS was 4 at T0 and 2 at T12 (right nasal cavity. Patient B: TENPS was 3 at T0 and 1 at T12 (left nasal cavity)

In Fig. 2 we report the rhinofibroscopy findings at T0 and T12 in 2 EGPA patients (A and B). Patient A had already received 2 FESS treatments in the previous years: TENPS was 4 at T0 and 2 at T12. Similarly, patient B had received 4 FESS treatments: TENPS was 3 at T0 and 1 at T12.

**Fig. 3:**
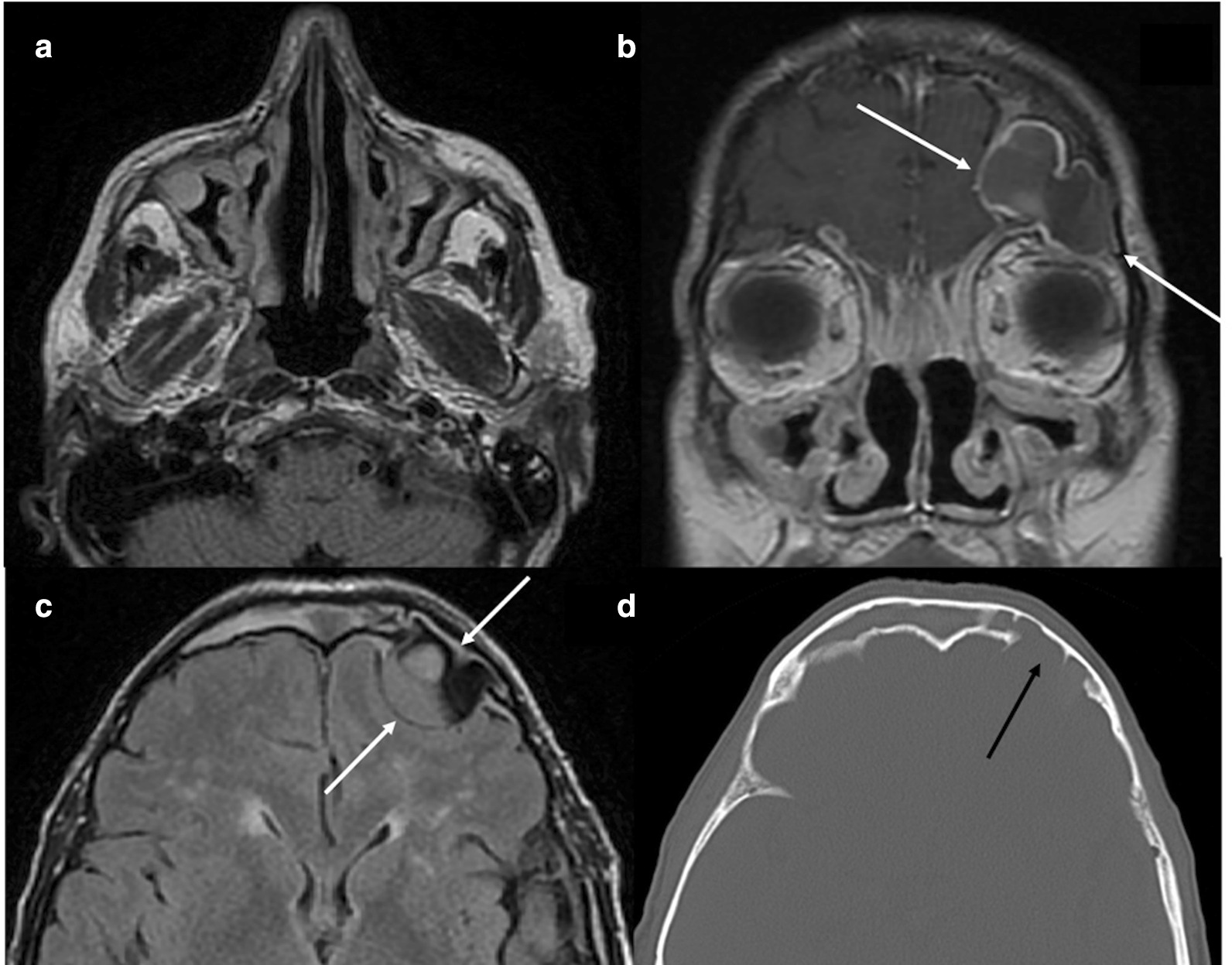
Axial T2-weighted MR image shows slightly hyperintense thickened mucosa in maxillary sinuses with concomitant retained secretions bilaterally (**a**). Preoperative coronal T1-weighted gadolinium enhanced MR scan. A multiloculated in homogeneously enhancing lesion protrudes from the left frontal sinus to the left frontal region, corresponding to a large mucocele (white arrows). Findings of superior and middle turbinectomy as well as bilateral maxillary antrostomy may be clearly depicted with slightly hyperintense thickened mucosa in both maxillary sinuses (**b**). Axial FLAIR MR sequence. A multiloculated lesion corresponding to a large mucocele, protrudes to the left frontal subarachnoid space, abutting the left frontal lobe (white arrows). The lesion shows internal areas of variable signal intensity, ranging from high to low (**c**). Brain CT in axial plane, bone window setting. The large mucocele causes an extensive erosion of the posterior wall of the left frontal sinus, protruding intracranially (black arrow) (**d**)

Another patient recruited for the study had severe recalcitrant nasal polyposis with a large left frontal sinus mucocele at T0 (TENPS 8). At the Magnetic Resonance Imaging (MRI) and computer tomography scan there was evidence of a mucocele protruding from the left frontal sinus to the left frontal region causing an extensive erosion of the posterior wall of the frontal sinus (Fig. 3a–d). The patient underwent sinus surgery in addition to FESS (5^th^ revision) during the study, while he was on mepolizumab treatment (after T6). This patient was excluded from SNOT-22 and TENPS evaluation at T12.

An overall clinical improvement in terms of asthma control was measured: mean ACT was 18.4 ± 8.9 at T6 and 20.4 ± 5.8 at T12 (T0- T12 p ≤ 0.01) (Fig. 1e). Mean % FEV1 was 71.2 ± 20.5 at T6 and 74.9 ± 21.8 at T12 (T0–T12, p = 0.78). In two patients, mepolizumab treatment had a poor efficacy in terms of asthma control: thus, these patients were switched to anti-IL-5R treatment (benralizumab) (one patient switched at T6 and the other at T12). Therefore, a total of 7 patients were included at T12 evaluation. Overall assessment was performed only on patients under mepolizumab treatment.

As expected, a drastic drop of eosinophil blood count was registered (mean eosinophils were 170.4 ± 100.5 cells/µl at T6 and 132.3 ± 152.4 cells/µl cells at T12; (T0 to T12, p < 0.01) (Fig. 1f).

Mepolizumab was well tolerated and no discontinuation due to severe adverse drug effects was registered. Most frequent adverse events were arthralgia (4/8), upper respiratory tract infection (3/8), worsening of asthma (2/8). As arthralgia may be secondary to adrenal suppression axis during OCS tapering, we performed assessment of hypothalamic–pituitary–adrenal axis integrity in all patients, according to the standard of care in order to exclude such correlation [36].

## Discussion

Therapeutic approach of EGPA is challenging for clinicians, as the standard of care treatments (systemic corticosteroids, immunosuppressive drugs) are hardly effective, and long-term remission is difficult to achieve in many patients. As EGPA is a rare disease, only a small number of randomized control trials (RCT) is available and there is a large need for real-life evidence. EGPA and SEA exhibit some similarities in their pathogenesis and therefore mepolizumab has been effectively used as the first targeted treatment in EGPA patients. However, it is still not clear whether the efficacy of the drug in the treatment of EGPA is due to its ability to control respiratory (asthma and sinonasal) symptoms or if it effectively plays a role in systemic vasculitis manifestations. As eosinophilia is central to the physio-pathogenesis of the disease, it is hypothesized that direct or indirect depletion of eosinophils may be beneficial for EGPA treatment. IL-5, is increased in patients with active EGPA and mediates different pathogenetic pathways leading to eosinophilic inflammation and tissue injury by promoting eosinophil adhesion to vascular endothelium and CC chemokine receptor 3 dependent migration of eosinophils from the vasculature [15]. Therefore, IL-5 blockage leads to eosinophilic reduction.

In our study, the mean BVAS at the time of mepolizumab initiation was 21 ± 8, reflecting an active disease. As BVAS is designed to document clinical features directly due to active vasculitis a significant BVAS reduction at T6 and T12 suggests an overall clinical improvement of the disease during mepolizumab treatment.

ENT involvement in EGPA is very frequent: rhinosinusitis, epistaxis, and neurosensory hearing loss are common features. Moreover, nasal polyposis may require recurrent surgical revisions [37]. There is real-life evidence of mepolizumab effectiveness in nasal polyposis and SEA [38, 39]. In the present study, mepolizumab treatment induced a significant reduction in SNOT-22 score (Fig. 1c). TENPS was also reduced, meaning that mepolizumab may control polyp growth in these patients (Figs. 1d, 2). 4 out of 8 patients had active polyposis at T0 previously treated with one or more FESS. One of these patients had a surgical removal of mucoceles during mepolizumab treatment. Mucoceles are inflammatory changes of the sinus mucosa that often occur following endoscopic sinus surgery for nasal polyposis [40, 41]. As mepolizumab may control polyp growth and reduce the need for FESS, mucoceles formation may be limited in patients under this treatment.

The efficacy of mepolizumab in SEA has been demonstrated in several studies: the drug induces reduction of asthma exacerbations and improvements in lung function (e.g., % FEV1) [20] both in RCT and real life studies [42, 43]. In this study, a rapid and steady increase in ACT score was observed in 6 patients. Interestingly, this clinical benefit was not paralleled by a significant increase in % FEV1 measurements.

Blood eosinophilia was sharply reduced. Apparently, this result is directly associated to the blocking effect of mepolizumab on circulating IL-5. The first clinical evidence of successful use of mepolizumab in EGPA was reported in 2010 by two different studies treating EGPA patients with 750 mg of monthly mepolizumab infusions [24, 41]. These studies reported a decreased disease activity and a safe OCS reduction during mepolizumab treatment. Wechsler et al. [19] demonstrated that administration of 300 mg of mepolizumab s.c. induced remission in approximately one half of EGPA patients. Moreover, real life evidence of 300 mg s.c. mepolizumab was recently reported by Ueno et al. [44] in active EGPA patients demonstrating decreased disease activity, increased remission rate and OCS sparing effect.

Experts suggest that the definition of complete response during EGPA treatment should include a prednisone dosage ≤ 7.5 mg/day to control systemic manifestations [45]. In this study, mean prednisone dosage was 7.1 ± 9.5 mg/day after 6 months of mepolizumab treatment. Among the non-responders one patient had persistent asthma and sinonasal respiratory symptoms under 7.5 mg/day of prednisone. The other patient had an initial improvement of respiratory symptoms followed by disease flare. Interestingly, both patients reported a sharp reduction of eosinophilia compared to T0 (T0 > 3000 vs T12: 200 cells/μL) that did not correlate to asthma (ACT) and sinonasal (SNOT-22, TENPS) improvement suggesting a non-eosinophilic pathogenetic pathway.

The most important target in the treatment of EGPA is to induce long-term remission and reduce the burden of systemic glucocorticoids and immunosuppressive therapies. In our study, 4 out of 8 patients had previously received or were on immunosuppressive drugs for disease control, and 7 patients were on prednisone at T0. Mepolizumab allowed corticosteroid tapering, exerting a corticosteroid-sparing effect. Given the side effects of systemic corticosteroid treatment in EGPA patients (weight gain, adrenal suppression, increased risk of infections, diabetes, osteoporosis, renal impairment, depression/anxiety), we suggest that reduction of prednisone intake in our patients may represent a main clinical benefit of mepolizumab treatment. In addition, during the observation period, patients remained clinically stable from the cutaneous, cardiologic, renal, and neurologic point of view. Mepolizumab treatment was safe, and no severe adverse events were registered. Upper respiratory tract infections or worsening of asthma could be part of the EGPA clinical picture and therefore could be considered as hallmarks of incomplete response to low-dose mepolizumab.

Recent real-life studies report evidence that low-dose mepolizumab can induce clinically relevant benefits in patients with non-active EGPA, especially on asthma symptoms and exacerbation rates, reduce the use of OCS and immunosuppressive drugs, and even prevent EGPA relapse for extrapulmonary manifestations [25–27]. Moreover, a case series by Vergles et al. [46], reported reasonable clinical efficacy in refractory and/or relapsing EGPA in 3 patients. Our study performed in patients with active EGPA may represent an interesting addition to the currently available data as low-dose mepolizumab effectiveness on BVAS, sinonasal outcomes (TENPS, SNOT-22), asthma outcomes (FEV1%, ACT), as well as OCS tapering and blood eosinophil count, have not been addressed together in previous reports in a real-life setting.

The study has some limitations: the open-label design, the absence of a control arm and the small number of participants make it difficult to extend our results to EGPA patients overall. Moreover, we prescribed low-dose (100 mg q4w) mepolizumab for severe eosinophilic asthma in our EGPA patients as high dose mepolizumab (300 mg q4w) is not yet approved for EGPA in Italy. However, these preliminary results can be a starting point for larger real-life studies, to define the most suitable, disease-severity tailored dosages of mepolizumab in EGPA patients.

## Data Availability

The data reported in this study are available from the corresponding author upon reasonable request.

## Abbreviations

EGPA: Eosinophilic granulomatosis with polyangiitis
SEA: Severe eosinophilic asthma
CRSwNP: Chronic Rhino-sinusitis with Nasal Polyps
CRSsNP: Chronic Rhino-sinusitis without Nasal Polyps
AEs: Adverse events
IL-5: Interleukin-5
OCS: Oral corticosteroids
BVAS: Birmingham Vasculitis Activity Score
SNOT-22: Sino-Nasal Outcome Test Score
TENPS: Total Endoscopic Polyp Score
ACT: Asthma control test
FEV1%: Forced expiratory volume at 1s%
